# Is Hysterosalpingography using Magnetic Resonance Imaging a promising tool in infertility patients?

**DOI:** 10.5935/1518-0557.20210104

**Published:** 2022

**Authors:** Ankita Sethi, Neeta Singh, Garima Patel

**Affiliations:** 1 Division of Reproductive Medicine, Department of Obstetrics and Gynaecology, All India Institute of Medical Sciences, New Delhi

Dear Editor

We read the recently published paper in your journal with great interest (Mattos *et al*., 2021). HSG-MRI can be a promising method for infertility evaluation. Although the study provides new insights into the HSG-MRI technique, as the reader, we would like to express some concerns based on the study analysis:

1. TVS is the preferred investigation method for a baseline assessment of ovarian and uterine pathology during the infertility workup (Vickramarajah *et al*., 2017; Practice Committee of the American Society for Reproductive Medicine, 2015). Ludwin *et al*. (2013) reported that 3D USG has 100% diagnostic accuracy to diagnose uterine anomalies when compared with the gold standard of laparoscopy and hysteroscopy. Similarly, 3D-USG is comparable to MRI to detect uterine anomalies and to visualize extrauterine structures (Pleş *et al*., 2018).

2. HYCOSY/Sonosalpingography can assess tubal patency as well as XR-HSG, thus eliminating the need for XR-HSG/MRI-HSG (Luciano *et al*., 2014). 

3. The article does not mention the indications for MRI

for the infertile couples recruited. This should be discussed, because of the limited indication of MRI in infertile couples. As mentioned above, most pelvic pathologies are detectable in USG, which is easily available in all clinical settings.

4. There was no disagreement concerning tubal patency, thus suggesting that MRI-HSG is not worse than XR-HSG. Therefore, MRI-HSG can only be done for infertile couples undergoing MRI for some other indication.

5. Fertiliscan combines HyFoSy with a high-quality 3D-USG for detailed infertility evaluation (Levaillant *et al*., 2019). Fertiliscan evaluates the uterine cavity, the adnexa, the ovarian reserve and fallopian tube patency. This has more advantages when compared to MRI-HSG, since it is more cost effective, it can be performed in the office setting; USG is more commonly available and accessible as compared to MRI in low resource settings, and in developing countries. Fertiliscan helps assess antral follicle count but not the MRI. So, this is a one stop investigation for infertility workup.

6. Thus, we need further studies to compare Fertiliscan with MRI-HSG, to see their advantages, disadvantages and clinical application.
